# Long-term survival in stroke patients: insights into triglyceride-glucose body mass index from ICU data

**DOI:** 10.1186/s12933-024-02231-0

**Published:** 2024-04-25

**Authors:** Yongwei Huang, Zongping Li, Xiaoshuang Yin

**Affiliations:** 1 Department of Neurosurgery, Mianyang Central Hospital, School of Medicine, University of Electronic Science and Technology of China, Mianyang, Sichuan China; 2 Department of Immunology, Mianyang Central Hospital, School of Medicine, University of Electronic Science and Technology of China, Mianyang, Sichuan China

**Keywords:** Triglyceride glucose-body mass index, Stroke, Mortality, Intensive care unit, MIMIC database

## Abstract

**Background:**

The Triglyceride Glucose-Body Mass Index (TyG-BMI) has been established as a robust indicator of insulin resistance (IR), reflecting metabolic health across various populations. In general, lower TyG-BMI values are often associated with better metabolic health outcomes and a reduced risk of adverse health events in non-critically ill populations. Previous studies have highlighted a significant negative association between TyG-BMI and all-cause mortality (ACM) among critically ill atrial fibrillation patients. Given the high prevalence and severe outcomes associated with stroke, understanding how TyG-BMI at the time of ICU admission correlates with ACM in critically ill stroke patients becomes imperative. This study aims to assess the correlation between TyG-BMI and ACM in this specific patient cohort, exploring how traditional associations between TyG-BMI and metabolic health may differ in the context of acute, life-threatening illness.

**Methods:**

Patient data were retrieved by accessing the Medical Information Mart for Intensive Care IV (MIMIC-IV 2.2) database, categorizing patients into three groups on the basis of TyG-BMI tertiles. The study evaluated both primary and secondary outcomes: the primary outcomes included the 90-day, 180-day, and 1-year ACM, while secondary outcomes encompassed ICU, in-hospital, and 30-day ACM. Our study employed the Kaplan–Meier (K–M) curve method for outcome comparison across the groups while utilizing multivariate Cox proportional-hazards regression models and restricted cubic splines (RCS) to explore TyG-BMI association with these outcomes. Additionally, interaction and subgroup analyses were performed, focusing on different mortality time points.

**Results:**

Among a cohort of 1707 individuals diagnosed with stroke, the average age was 68 years (interquartile range [IQR]: 58–78 years), with 946 (55.42%) of the participants being male. The analysis of K-M curves suggested that patients having a lower TyG-BMI level faced a heightened risk of long-term ACM, whereas the short-term ACM exhibited no statistically significant differences across the three TyG-BMI groups. Furthermore, Cox proportional-hazards regression analysis validated a statistically significant increased risk of long-term ACM among patients belonging to the lowest TyG-BMI tertile. Additionally, RCS analysis results demonstrated L-shaped correlations between the TyG-BMI index and both short- and long-term ACM. These findings underscore the TyG-BMI predictive value for long-term mortality in stroke patients, highlighting a nuanced relationship that varies over different time frames. The results revealed no interactions between TyG-BMI and the stratified variables, with the exception of age.

**Conclusion:**

In our study, lower TyG-BMI levels in critically ill stroke patients are significantly related to a higher risk of long-term ACM within the context of the United States. This finding suggests the potential of TyG-BMI as a marker for stratifying long-term risk in this patient population. However, it's crucial to note that this association was not observed for short-term ACM, indicating that the utility of TyG-BMI may be more pronounced in long-term outcome prediction. Additionally, our conclusion that TyG-BMI could serve as a reliable indicator for managing and stratifying stroke patients over the long term is preliminary. To confirm our findings and assess the universal applicability of TyG-BMI as a prognostic tool, it is crucial to conduct rigorously designed research across various populations.

**Supplementary Information:**

The online version contains supplementary material available at 10.1186/s12933-024-02231-0.

## Introduction

Epidemiological studies have indicated that cerebrovascular disease (CVD) rendered it the primary contributor to fatality and disability in American adults except for cardiovascular diseases [1, 2]. As the CVD predominant component, the incidence of stroke is on the rise, marking it as a significant global health issue responsible for substantial mortality and long-term disability [3]. Strokes are primarily categorized into ischemic and hemorrhagic types. Ischemic strokes, which constitute around 85% of all stroke cases, occur due to obstructions in the arteries that supply blood to the brain, often owing to thrombosis or embolism [4]. Hemorrhagic stroke, conversely, involves bleeding into or around the brain, usually because of weakened blood vessel rupture and includes subtypes such as subarachnoid hemorrhage (SAH) and non-traumatic intracerebral hemorrhage (ICH). ICH experiences a yearly rise of over 3.41 million instances [5], whereas SAH represents 5% of all strokes [6]. Both ischemic and hemorrhagic strokes lead to high mortality rates and extensive disabilities [7–9]. The increasing worldwide population aging causes a substantial stroke burden, especially in intensive care units (ICU). Thus, identifying prognostic indicators that can foresee adverse outcomes in stroke patients is of paramount importance. These indicators should be straightforward, user-friendly, cost-efficient, and readily applicable in clinical settings.

Stroke severity, a critical determinant of patient outcomes, is traditionally assessed using scales such as the NIH Stroke Scale and the Canadian Neurological Scale. These tools, while invaluable, have limitations, including complexity, time consumption, and the need for specialized training. In the search for more accessible and efficient prognostic tools, insulin resistance (IR) has been related to stroke [10] and is characterized by the body's compensatory excessive insulin production to counteract diminished insulin efficacy in glucose uptake and utilization, leading to hyperinsulinemia to maintain normoglycemia. IR represents a characteristic feature of diabetes Mellitus (DM) type 2 and metabolic syndrome [11]. The hyperinsulinemic-euglycemic clamp (HEC) represents the gold standard for IR assessment across various populations [12]. However, the homeostasis model assessment-estimated IR (HOMA-IR) index, a reliable IR measure, necessitates specialized equipment, trained personnel, substantial expenses, an extended period, and several blood samples, besides being less patient-friendly. Due to these limitations, HOMA-IR is mainly confined to research settings and not widely adopted in clinical practice due to its operational complexities and bio-efficacy concerns.

The triglyceride-glucose index (TyG-i) has recently been a straightforward surrogate IR marker. Prior research has validated its predictive capacity for cardiovascular disease outcomes that included acute myocardial infarction (AMI), coronary heart disease (CHD), heart failure (HF), stroke, and hypertension (HTN) [11, 13–16]. Notably, integrating the TyG-i along with obesity measures, including body mass index (BMI), waist circumference (WC), and waist-to-height ratio (WtHR), has significantly enhanced IR assessment accuracy [16]. The combined TyG-BMI, particularly, has shown promising alignment with HOMA-IR in evaluating IR [17] and is closely linked to HTN prevalence, non-alcoholic fatty liver disorder, and cardiovascular outcomes in coronary artery disease patients [18–20]. Based on the study of TyG-BMI and mortality in critically ill atrial fibrillation patients [21], We hypothesis that a significant negative association between TyG-BMI and ACM among critically ill stroke patients still existed. While the TyG-BMI's positive association with stroke risk in the Chinese general population has been documented [22–24], its impact on outcomes for stroke patients in the ICU remains underexplored. Given the high prevalence and severe outcomes associated with critical ill stroke patients, understanding how TyG-BMI at the time of ICU admission correlates with all-cause mortality (ACM) in critically ill stroke patients becomes imperative. This study aims to assess the correlation between TyG-BMI and ACM in this specific patient cohort, exploring how traditional associations between TyG-BMI and metabolic health may differ in the context of acute, life-threatening illness. The findings could offer new avenues for the early identification and prognostic improvement of critically ill stroke patients, addressing a significant gap in current clinical practice and research.

## Methods

### Source of data

This retrospective study used data sourced from the publicly accessible Medical Information Mart for Intensive Care IV database (MIMIC-IV, version 2.2), which is an enhancement of its predecessor, MIMIC-III, that features updated data and some structural modifications to tables. The dataset includes clinical information from over 190,000 unique patient admissions at Beth Israel Deaconess Medical Center (BIDMC; Boston, MA, USA) from 2008 to 2019. The database provides comprehensive details on patient demographics, vital statistics, medications, laboratory tests, surgical procedures, diagnoses, treatment regimens, and survival outcomes. To access this data, we completed the National Institutes of Health (NIH) training course on the protection of human research participants and passed the Collaborative Institutional Training Initiative's tests. An informed consent waiver was obtained because the database does not contain any protected health information, and the patients remain anonymous.

### Study design and population

**Fig. 1:**
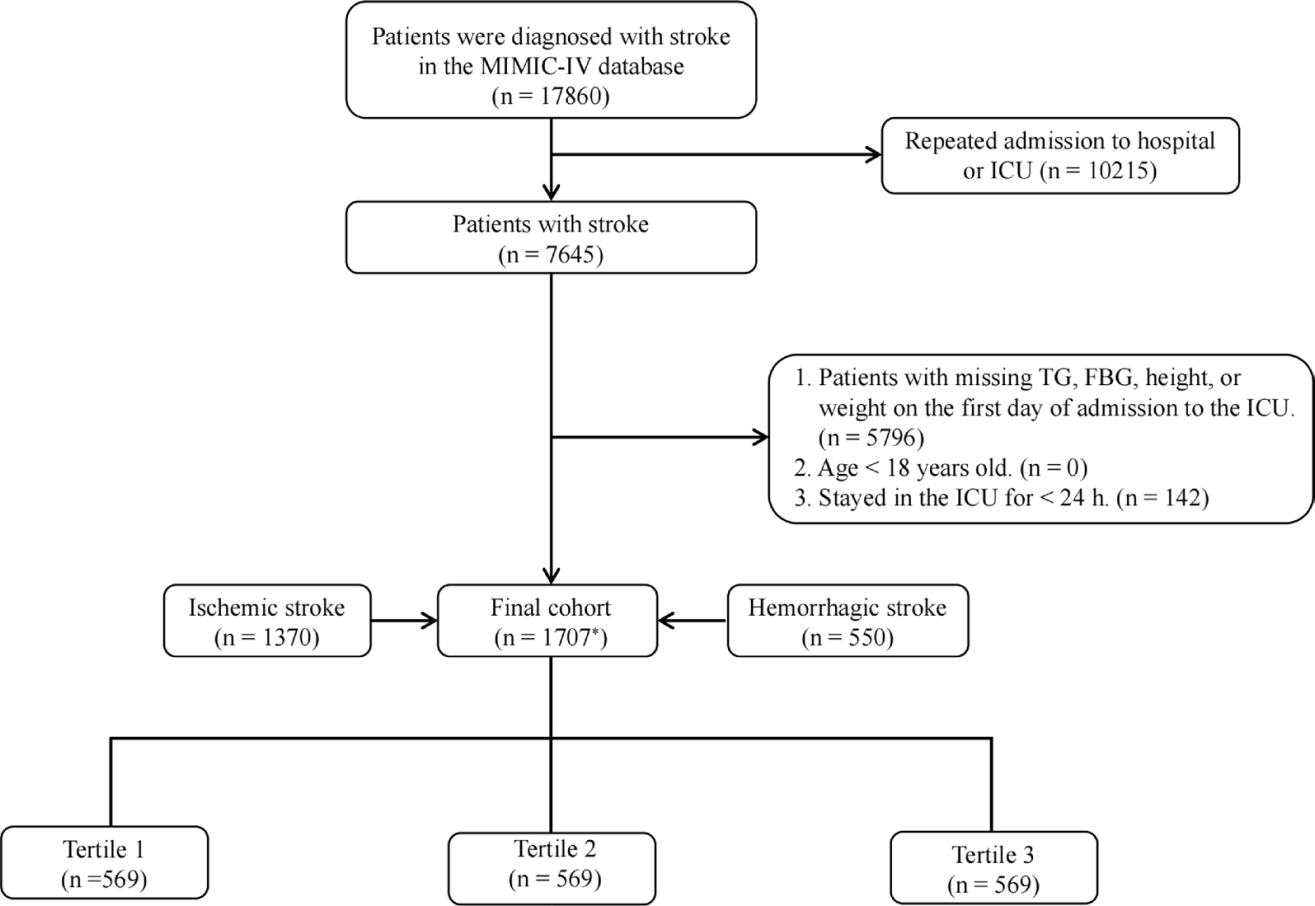
The study flowchart. *There are 213 patients diagnosed with both ischemic and hemorrhagic stroke. MIMIC-IV: Medical Information Mart for Intensive Care IV; ICU: intensive care unit; TG: triglycerides: FBG: fasting blood glucose

The study focused on patients who experienced a stroke and were subsequently hospitalized and admitted to the ICU for the first time. We identified a cohort of 17,860 stroke patients using the search terms “non-traumatic ICH,” “non-traumatic SAH,” and “cerebral infarction” within the International Classification of Diseases, Ninth (ICD-9) and Tenth Revision (ICD-10) diagnostic codes. The exclusion criteria were established to ensure data accuracy and relevance: (1) absence of data on triglycerides (TG), fasting blood glucose (FBG), height, or weight on first-day ICU admission; (2) patients younger than 18 years; (3) ICU stays shorter than 24 h; and (4) patients experienced multiple ICU admissions for stroke, with only the first admission’s data being considered. Following these criteria, 1,707 patients were selected and categorized into three groups depending on TyG-BMI tertiles for further analysis (Fig. 1).

### Data extraction

Data extraction was performed using Navicat Premium (Version 16.1.15) employing structured query language (SQL). Comprehensive data regarding each patient at admission time were collected, such as demographic variables: gender, age, height (m), weigh (kg), and ethnicity; clinical severity scores: baseline Glasgow Coma score, sequential organ failure assessment (SOFA) score, simplified acute physiology score (SAPS)-II, acute physiology (APS) score-III, oxford acute severity of illness (OASIS) score, systemic inflammatory response syndrome (SIRS) score; baseline vital signs: the mean blood pressure (mmHg), mean heart rate (beats/min), systolic blood pressure (SBP, mmHg), respiratory rate (times/min), diastolic blood pressure (mmHg), and saturation of pulse oxygen (SPO_2_, %); Laboratory variables: red blood cell (RBC, 10^9^/L), hemoglobin (Hb, g/L), platelet (10^9^/L), white blood cell (WBC, 10^9^/L), sodium (mmol/L); blood urea nitrogen (BUN, mg/dL); creatinine (mg/24 h); triglyceride (TG, mg/dL); fasting blood glucose (FBG, mg/dL); potassium (mmol/L), anion gap (mmol/L), prothrombin time (s), activated partial thromboplastin time (s); international normalized ratio; treatment methods: mechanical ventilation (MT), vasopressors, oxygen, thrombolysis, thrombectomy; comorbidities: HTN, DM, HF, cardiac arrhythmias, AMI, peripheral vascular disease, chronic obstructive pulmonary disease, respiratory failure, ventilator associated pneumonia (VAP), chronic kidney disease, hyperlipidemia, malignancy, renal failure, sepsis, liver disease, Charlson comorbidity index; outcomes: Length of stay (LOS) in ICU, LOS in hospital; ICU, in-hospital, 30-day; 90-day, 180-day, 1-year ACM. All blood indicators were recorded at the first measurement post-ICU admission. The dataset had no variables exceeding 20% missing values, and any missing data were imputed using multiple interpolation techniques.

### Outcomes

The study focused on assessing the ACM outcomes for stroke patients within the MIMIC-IV database at various intervals. The primary endpoints were designated as long-term ACM, which included assessments at 90-day, 180-day, and 1-year post-admission. The secondary outcomes encompassed short-term ACM metrics, such as mortality within the ICU, in-hospital, and 30-day periods following admission.

### TyG-BMI calculation

The TyG-i was calculated as follows: Ln[FBG (mg/dL) × TG (mg/dL)/2] while calculating BMI by dividing the body weight (kg) by the square of height (m) (kg/m^2^). The TyG-BMI, a composite metric, was then derived by multiplying the TyG-i by the BMI, according to the formula: TyG × BMI. This combined measure was used for the correlation assessment between metabolic status and outcomes in stroke patients.

### Statistical analysis

Initial testing was performed to assess the normality of continuous variables. Student t-tests and one-way ANOVAs were utilized for normally distributed data, expressing the results as mean ± standard deviation (SD). For non-normally distributed data, the Wilcoxon rank-sum test was applied, with findings reported as medians alongside interquartile ranges (IQR). Moreover, we analyzed categorical variables through Chi-square or Fisher’s exact tests, reporting data as absolute numbers and percentages.

To evaluate the primary and secondary outcome incidence rates based on TyG-BMI stratification, we employed Kaplan–Meier (K-M) curves. Univariable Cox regression analysis was deployed to explore the relationships between TyG-BMI and both short- and long-term ACM. The multivariate Cox proportional-hazard regression model was then utilized, incorporating variables of clinical relevance or those demonstrating a univariate association with outcomes. The final model variables were selected considering the availability of event data. Model 1 was unjusted while Model 2 adjusted for age, gender, and ethnicity and Model 3 further adjusted for HTN, DM, HF, thrombolysis, thrombectomy, WBC, RBC, SBP, and SOFA score, using the lowest TyG-BMI tertile as the reference. Furthermore, TyG-BMI was examined as a continuous variable through restricted cubic splines (RCS) to elucidate dose–effect relationships with primary and secondary outcome risk. In cases of nonlinear correlations, a recursive algorithm determined the inflection points between TyG-BMI and short- and long-term ACM. This study conducted stratified analyses on the basis of age (< 60 or ≥ 60 years old), sex, race/ethnicity, HTN, and DM. Statistical analyses were performed through SPSS (version 22.0, IBM Corporation, United States) and R software (version 4.3.2, R Foundation for Statistical Computing, Austria), with P < 0.05 considering statistical significance.

## Results

### Baseline characteristics of study individuals

Herein, out of 17,860 stroke patients listed in the MIMIC-IV database, 1,707 met the inclusion criteria and were subsequently analyzed. They had an average age of 68 years, with an IQR of 58–78 years, and 55.4% of them were male. Our study categorized the participants into tertiles relying on their TyG-BMI upon admission: Tertile 1 (< 224.6), Tertile 2 (224.6–276.1), and Tertile 3 (> 276.1). Baseline characteristics (Table 1) revealed varying TyG-BMI levels across the groups: 196.6 for Tertile 1 (IQR: 175.8–210.3), 248.1 for Tertile 2 (IQR: 236.4–261.3), and 323.1 for Tertile 3 (IQR: 296.4–369.7).

**Table 1:** The baseline characteristics and outcomes of participants classified by triglyceride glucose-body mass index (TyG-BMI) tertiles

Variable	Overall (n = 1707)	Tertile 1 (n = 569)	Tertile 2 (n = 569)	Tertile 3 (n = 569)	*P* value
TyG-BMI	248.1 (210.3–296.4)	196.6 (175.8–210.3)	248.1 (236.4–261.3)	323.1 (296.4–369.7)	< 0.001
Demographics					
Age, years	68 (58–78)	72 (61–81)	69 (59–78)	65 (55–73)	< 0.001
Men, n (%)	946 (55.42)	301 (52.90)	329 (57.82)	316 (55.54)	0.25
Race/ethnicity, n (%)					< 0.001
Asian	56 (3.28)	33 (5.80)	19 (3.34)	4 (0.70)	
White	190 (11.13)	58 (10.19)	68 (11.95)	64 (11.25)	
Black	1015 (59.46)	338 (59.40)	335 (58.88)	342 (60.11)	
Others	446 (26.13)	140 (24.60)	147 (25.83)	159 (27.94)	
Comorbidities					
Hypertension, n (%)	923 (54.07)	296 (52.02)	316 (55.54)	311 (54.66)	0.46
Diabetes mellitus, n (%)	563 (32.98)	121 (21.27)	171 (30.05)	271 (47.63)	< 0.001
Heart failure, n (%)	473 (27.71)	161 (28.30)	147 (25.83)	165 (29.00)	0.46
Cardiac arrhythmias, n (%)	784 (45.93)	266 (46.75)	257 (45.17)	261 (45.87)	0.87
Acute myocardial infarction, n (%)	28 (1.64)	8 (1.41)	11 (1.93)	9 (1.58)	0.78
Peripheral vascular disease, n (%)	160 (9.37)	56 (9.84)	51 (8.96)	53 (9.32)	0.88
Chronic obstructive pulmonary disease, n (%)	118 (6.91)	38 (6.68)	32 (5.62)	48 (8.44)	0.17
Respiratory failure, n (%)	676 (39.6)	222 (39.0)	206 (36.2)	248 (43.6)	0.04
Chronic kidney disease, n (%)	340 (19.92)	98 (17.22)	114 (20.04)	128 (22.50)	0.08
Hyperlipidemia, n (%)	959 (56.18)	270 (47.45)	349 (61.34)	340 (59.75)	< 0.001
Malignancy, n (%)	320 (18.75)	137 (24.08)	102 (17.93)	81 (14.24)	< 0.001
Renal failure, n (%)	1369 (80.20)	397 (69.77)	453 (79.61)	519 (91.21)	< 0.001
Sepsis, n (%)	1108 (64.91)	353 (62.04)	367 (64.50)	388 (68.19)	0.09
Ventilator-associated pneumonia, n (%)	165 (9.67)	38 (6.68)	65 (11.42)	62 (10.90)	0.01
Liver disease, n (%)	234 (13.71)	70 (12.30)	82 (14.41)	82 (14.41)	0.49
Charlson comorbidity index	6 (4–8)	6 (4–8)	6 (4–8)	6 (4–7)	0.005
Baseline vital signs					
Height, m	1.68 (1.60–1.78)	1.68 (1.60–1.75)	1.68 (1.60–1.78)	1.68 (1.60–1.78)	0.15
Weight, kg	78.4 (65.2–93.9)	62.0 (54.5–70.5)	78.0 (70.4–86.4)	100.0 (86.9–114.0)	< 0.001
Body mass index, kg/cm^2^	27.4(23.9–32.2)	22.4 (20.3–24.2)	27.6 (26.1–29.1)	34.7 (32.0–38.6)	< 0.001
Mean blood pressure, mmHg	86 (75–100)	86 (75–98)	88 (75–102)	86 (76–100)	0.19
Systolic blood pressure, mmHg	129 (113–149)	128 (112–147)	131 (114–152)	129 (113–147)	0.14
Diastolic blood pressure, mmHg	69 (58–83)	68 (58–81)	71 (59–84)	69 (59–85)	0.23
Mean heart rate, beats/min	82 (72–96)	83 (71–96)	81 (72–95)	83 (73–96)	0.62
Respiratory rate, times/min	18 (15–22)	18 (15–21)	18 (15–22)	18 (15–22)	0.31
Saturation of pulse oxygen, %	99 (96–100)	99 (97–100)	99 (96–100)	98 (96–100)	< 0.001
Laboratory parameters					
Red blood cell, 10^9^/L	3.79 (3.20–4.32)	3.67 (3.12–4.21)	3.85 (3.22–4.31)	3.86 (3.25–4.40)	0.002
Hemoglobin, g/L	11.4 (9.6–13.0)	11.1 (9.4–12.7)	11.6 (9.7–13.1)	11.5 (9.6–13.1)	0.01
Platelet, 10^9^/L	191 (146–248)	193 (149–253)	185 (138–246)	195 (153–248)	0.14
White blood cell, 10^9^/L	11.0 (8.1–14.3)	10.8 (7.7–14.0)	10.9 (8.1–14.0)	11.7 (8.4–15.1)	0.008
Sodium, mmol/L	139 (136–141)	139 (136–142)	139 (137–141)	138 (136–141)	0.14
Blood urea nitrogen, mg/dL	18 (13–28)	18 (13–26)	18 (13–28)	19 (14–30)	0.02
Creatinine, mg/24 h	1.0 (0.7–1.4)	0.9 (0.7–1.3)	1.0 (0.8–1.3)	1.0 (0.8–1.5)	< 0.001
Triglyceride, mg/dL	117 (84–177)	91 (70–122)	118 (87–174)	157 (111–239)	< 0.001
Fasting blood glucose, mg/dL	130 (107–166)	118 (101–148)	129 (107–160)	144 (117–188)	< 0.001
Potassium, mmol/L	4.1 (3.7–4.5)	4.1 (3.7–4.5)	4.1 (3.7–4.5)	4.1 (3.7–4.5)	0.44
AG, mmol/L	14 (12–16)	14 (11–16)	13 (11–16)	14 (12–16)	0.10
Prothrombin time, s	13.4 (12.1–15.6)	13.4 (11.8–15.7)	13.4 (12.1–15.7)	13.4 (12.2–15.5)	0.63
Activated partial thromboplastin time, s	29.5 (26.3–34.7)	29.7 (26.7–35.2)	29.3 (26.3–34.9)	29.3 (26.1–33.8)	0.10
International normalized ratio	1.2 (1.1–1.4)	1.2 (1.1–1.4)	1.2 (1.1–1.4)	1.2 (1.1–1.4)	0.40
Clinical severity scores					
Baseline Glasgow Coma score	15 (15–15)	15 (14–15)	15 (15–15)	15 (15–15)	0.02
SOFA	1 (0–3)	1 (0–2)	1 (0–2)	1 (0–3)	0.29
SAPS-II	35 (28–45)	37 (29–45)	35 (28–45)	35 (28–44)	0.15
SIRS	3 (2–3)	3 (2–3)	3 (2–3)	3 (2–3)	0.37
OASIS	33 (27–38)	33 (28–38)	32 (27–38)	33 (28–39)	0.22
APS-III	41 (30–55)	42 (31–55)	40 (30–54)	40 (30–56)	0.10
Treatment					
Mechanical ventilation, n (%)	1483 (86.9)	467 (82.1)	495 (87.0)	521 (91.6)	< 0.001
Vasopressors, n (%)	735 (43.06)	226 (39.72)	240 (42.18)	269 (47.28)	0.03
Oxygen, n (%)	1331 (77.97)	417 (73.29)	442 (77.68)	472 (82.95)	< 0.001
Thrombolysis, n (%)	100 (5.86)	31 (5.45)	37 (6.50)	32 (5.62)	0.72
Thrombectomy, n (%)	192 (11.25)	60 (10.54)	65 (11.42)	67 (11.78)	0.79
Stroke type					
Ischemic stroke, n (%)	1370 (80.26)	449 (78.91)	455 (79.96)	466 (81.90)	0.44
Non-traumatic intracerebral hemorrhage, n (%)	434 (25.42)	150 (26.36)	154 (27.07)	130 (22.85)	0.22
Non-traumatic subarachnoid hemorrhage, n (%)	116 (6.80)	32 (5.62)	42 (7.38)	42 (7.38)	0.40
Clinical outcomes					
LOS ICU, day	5 (2–10)	4 (2–8)	5 (2–10)	6 (3–12)	< 0.001
LOS hospital, day	11 (6–20)	9 (6–19)	11 (6–20)	13 (7–21)	**0.003**
Short-term all-cause mortality					
ICU mortality, n (%)	211 (12.36)	68 (11.95)	69 (12.13)	74 (13.01)	0.85
In-hospital mortality, n (%)	300 (17.57)	101 (17.75)	97 (17.05)	102 (17.93)	0.92
30-day mortality, n (%)	354 (20.74)	136 (23.90)	111 (19.51)	107 (18.80)	0.07
Long-term all-cause mortality					
90-day mortality, n (%)	469 (27.48)	188 (33.04)	141 (24.78)	140 (24.60)	0.001
180-day mortality, n (%)	523 (30.64)	212 (37.26)	162 (28.47)	149 (26.19)	< 0.001
1-year mortality, n (%)	584 (34.21)	233 (40.95)	184 (32.34)	167 (29.35)	< 0.001

SOFA: sequential organ failure assessment score; SAPS-II: simplified acute physiology score; SIRS: systemic inflammatory response syndrome score; OASIS: oxford acute severity of illness score; APS-III: acute physiology score-III; LOS: length of stay; ICU: intensive care unit

The analysis indicated that the lowest TyG-BMI group (Tertile 1) participants were older and had lower weight, BMI, and prevalence rates of respiratory failure, hyperlipidemia, malignancy, sepsis, and VAP. They also showed lower utilization rates of MT, vasopressors, and oxygen therapy. Additionally, lower levels of RBC, Hb, WBC, BUN, creatinine, TG, and FBG, as well as shorter LOS in both the ICU and hospital, were observed in the Tertile 1 group. Conversely, this group exhibited higher levels of SpO_2_ than those with higher TyG-BMI levels. Regarding outcomes, the Tertile 1 group exhibited higher long-term ACM compared to the other two groups, while the short-term ACM did not significantly differ among the groups. For validity of the results, we also reported the baseline characteristics and outcomes of the excluded and included patients. More detailed results are presented in Additional file 1: Table S1.

### Primary outcomes

**Fig. 2:**
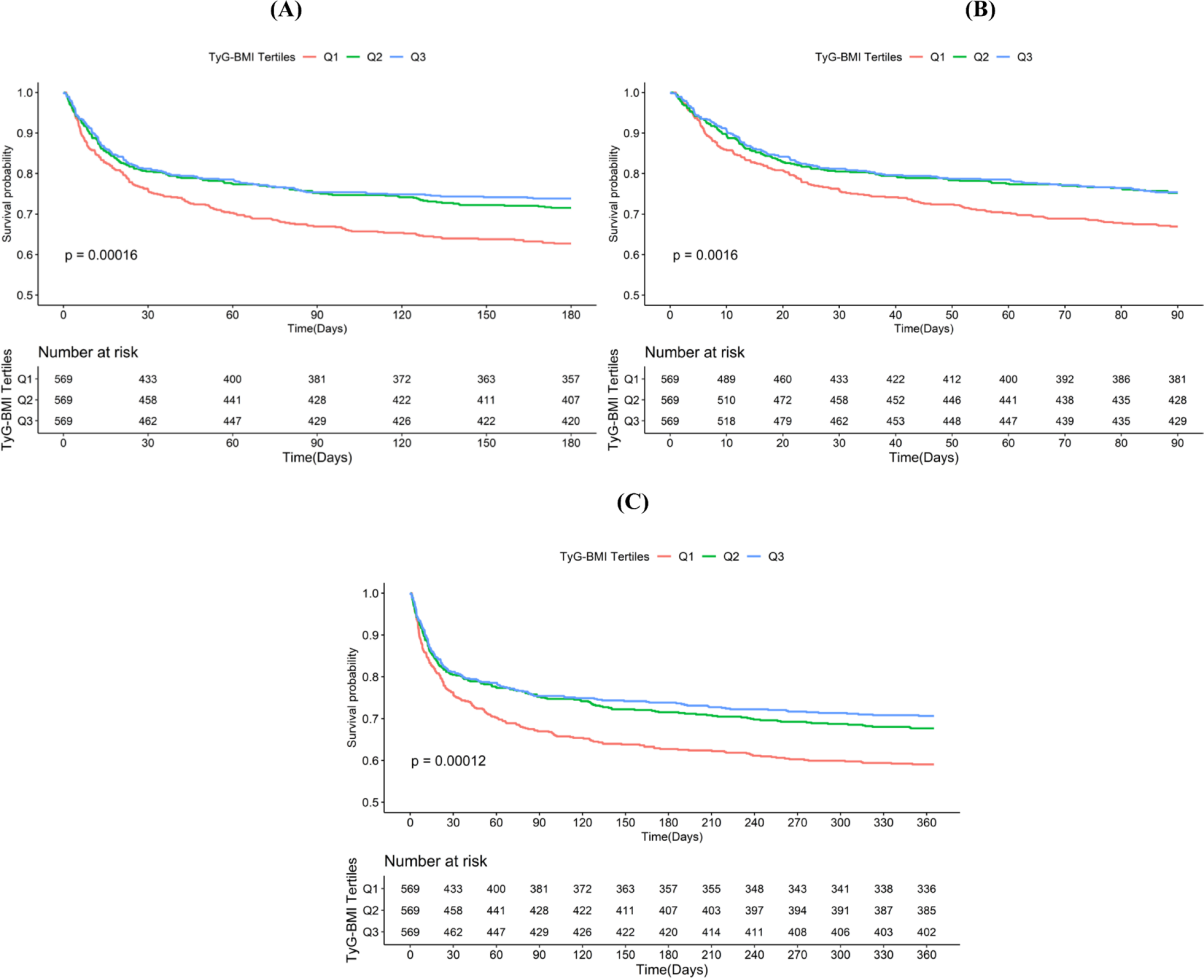
K-M survival analysis curves for ACM and cumulative incidence of 90-day (**A**), 180-day (**B**), and 1-year (**C**) ACM

The K-M analysis revealed varying ACM at 90-day, 180-day, and 1-year intervals across TyG-BMI tertiles (Fig. 2). Patients within the lowest TyG-BMI tertile exhibited significantly lower long-term survival rates than those with higher TyG-BMI levels, with log-rank P-values of 0.00016, 0.0016, and 0.00012, respectively. In contrast, differences in short-term ACM, including ICU, in-hospital, and 30-day mortality, among the three groups did not reach statistical significance, with log-rank P-values of 0.52, 0.68, and 0.067, respectively (Additional file 1: Fig. S1).

The clinical TyG-BMI utility for mortality prediction was determined through receiver operating characteristic (ROC) curves. However, the area under the curve (AUC) values indicated limited predictive power (ICU death AUC: 0.52; in-hospital death AUC: 0.50; 30-day death AUC: 0.53; 90-day death AUC: 0.55; 180-day death AUC: 0.56; 1-year death AUC: 0.55;) (Additional file 1: Fig. S2).

### Correlation between TyG-BMI and clinical outcomes of patients having a stroke

To explore the independent TyG-BMI impact on ACM, three Cox proportional hazard regression models were employed (Table 2), adjusting Model 1 for no adjustment, Model 2 for age, gender, and ethnicity, and Model 3 for Model 2 variables, HTN, DM, HF, thrombolysis, thrombectomy, WBC, RBC, SBP, and SOFA score. In Model 3, using the lowest TyG-BMI tertile (Q1) as the reference, the hazard ratios (HRs) and 95% confidence intervals (CIs) for Q2/Q3 were 0.74 (0.59–0.92) and 0.74 (0.59–0.94) for 90-day ACM, 0.74 (0.60–0.91) and 0.69 (0.55–0.86) for 180-day ACM, and 0.75 (0.61–0.91) and 0.67 (0.54–0.83) for 1-year ACM, respectively. These findings indicate that patients with lower TyG-BMI levels faced a higher long-term ACM risk than those with a TyG-BMI index ≥ 224.6. Nonetheless, the TyG-BMI and short-term ACM relationship exhibited no significant differences (Additional file 1: Table S1).

**Table 2:** Multivariable Cox proportional hazard models for long-term ACM

Long-term ACM	Model 1		Model 2		Model 3	
	HR (95% CI)	*P* value	HR (95% CI)	*P* value	HR (95% CI)	*P* value
90-day mortality						
TyG-BMI (tertiles)						
Tertile 1 (< 224.6)	Reference		Reference		Reference	
Tertile 2 (224.6–276.1)	0.72 (0.58–0.90)	0.003	0.74 (0.60–0.92)	0.008	0.74 (0.59–0.92)	0.007
Tertile 3 (> 276.1)	0.71 (0.57–0.88)	0.002	0.75 (0.60−0.93)	0.009	0.74 (0.59–0.94)	0.02
*P* for trend		0.006		0.02		0.04
180-day mortality						
TyG-BMI (tertiles)						
Tertile 1 (< 224.6)	Reference		Reference		Reference	
Tertile 2 (224.6–276.1)	0.73 (0.59–0.90)	0.002	0.74 (0.61–0.91)	0.005	0.74 (0.60–0.91)	0.005
Tertile 3 (> 276.1)	0.66 (0.54–0.82)	< 0.001	0.69 (0.56–0.86)	< 0.001	0.69 (0.55–0.86)	< 0.001
*P* for trend		< 0.001		0.003		0.005
1-year mortality						
TyG-BMI (tertiles)						
Tertile 1 (< 224.6)	Reference		Reference		Reference	
Tertile 2 (224.6–276.1)	0.74 (0.61–0.90)	0.003	0.76 (0.62–0.92)	0.005	0.75 (0.61–0.91)	0.004
Tertile 3 (> 276.1)	0.67 (0.55–0.82)	< 0.001	0.69 (0.57–0.85)	< 0.001	0.67 (0.54–0.83)	< 0.001
*P* for trend		< 0.001		0.002		0.002

Unadjusted Model 1;

Gender, age, and ethnicity-adjusted Model 2;

Gender, age, ethnicity, HTN, DM, HF, thrombolysis, thrombectomy, WBC, RBC, SBP, and SOFA-adjusted Model 3

### The detection of nonlinear relationships

**Fig. 3:**
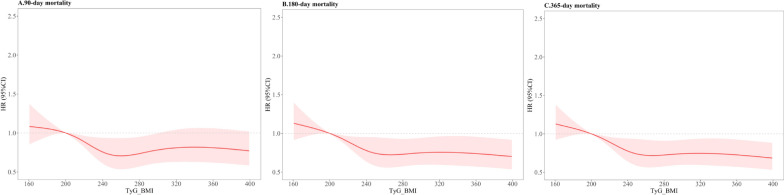
Restricted cubic spline analysis illustrating TyG-BMI with 90-day (**A**), 180-day (**B**), and 1-year (**C**) ACM

The RCS analyses revealed L-shaped correlations between TyG-BMI and long-term and short-term ACM, indicating a threshold effect beyond which the risk of mortality does not significantly change. Specifically, the RCS curves for long-term ACM showed significant non-linearity (P_nonlinearity_ = 0.16 for 90-day, P_nonlinearity_ = 0.09 for 180-day, P_nonlinearity_ = 0.09 for 1-year, depicted in Fig. 3A–C). Similarly, the short-term ACM analyses also demonstrated significant nonlinear relationships (P_nonlinearity_ = 0.82 for ICU, P_nonlinearity_ = 0.93 for in-hospital, P_nonlinearity_ = 0.34 for 30-day, illustrated in Additional file 1: Fig. S3A–C).

These findings suggest that beyond a certain level of TyG-BMI, the risk of both short- and long-term ACM plateaus, highlighting the significance of identifying and managing patients at risk based on their TyG-BMI levels. The exact points of non-linearity are critical for understanding the thresholds at which TyG-BMI begins to have a diminished impact on mortality risk, guiding clinical decisions regarding the monitoring and intervention for patients with stroke in both acute and long-term care settings.

### Subgroup analysis

**Fig. 4:**
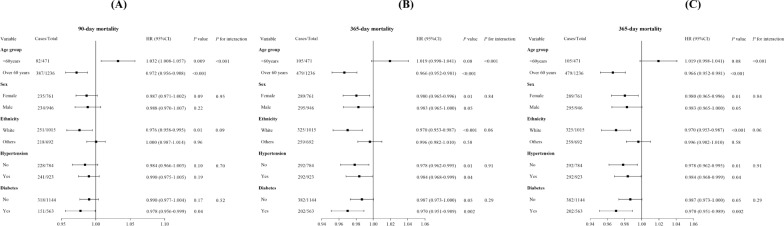
Forest plots illustrating stratified analyses of association of TyG-BMI and 90-day (**A**), 180-day (**B**), and 1-year (**C**) ACM

The TyG-BMI's risk stratification value for primary endpoints was further examined in multiple subgroups of the participants, including sex, age, ethnicity, HTN, and DM (Table 3**, **Fig. 4A–C). The TyG-BMI was significantly correlated with a lower mortality risk in stroke patients subgroups of those aged over 60 years for 90-day [HR (95% CI) 0.972 (0.956–0.988)], 180-day [HR (95% CI) 0.965 (0.949–0.980)], and 1-year [HR (95% CI) 0.966 (0.952–0.981)]. Additionally, TyG-BMI was related to a higher mortality risk in stroke patients subgroups of those aged under 60 years for 90-day [HR (95% CI) 1.032 (1.008–1.057)] and 180-day [HR (95% CI) 1.027 (1.005–1.051)]. Regarding sex, TyG-BMI was significantly correlated with a lower mortality risk in stroke patients subgroups of females for 180-day [HR (95% CI) 0.983 (0.968–0.999)], and 1-year [HR (95% CI) 0.980 (0.965–0.996)]. Regarding race/ethnicity, the TyG-BMI was significantly linked to a lower mortality risk in stroke patients subgroups of White for 90-day [HR (95% CI) 0.976 (0.958–0.995)], 180-day [HR (95% CI) 0.971 (0.953–0.989)], and 1-year [HR (95% CI) 0.970 (0.953–0.987)]. Concerning HTN, TyG-BMI had a significant relation to a lower mortality risk in stroke patients subgroups of non-HTN for 180-day [HR (95% CI) 0.978 (0.960–0.996)], both non-HTN and HTN for 1-year [HR (95% CI) 0.978 (0.962–0.995); 0.984 (0.969–0.999)]. Concerning DM, TyG-i showed a significant relation to a lower mortality risk in stroke patients subgroups of DM for 90-day [HR (95% CI) 0.978 (0.956–0.999)], 180-day [HR (95% CI) 0.970 (0.949–0.991)], and 1-year [HR (95% CI) 0.970 (0.951–0.989)]. Forest plots in Additional file 1: Fig. S4A–C depict the stratified analyses of TyG-BMI and short-term ACM.

**Table 3:** Subgroup analyses of TyG-BMI and long-term ACM

Variable	Cases/total	HR (95% CI)	*P* value	*P* for interaction
90-day mortality				
Age group				< 0.001
< 60 years	82/471	1.032 (1.008–1.057)	0.009	
Over 60 years	387/1236	0.972 (0.956–0.988)	< 0.001	
Sex				0.95
Female	235/761	0.987 (0.971–1.002)	0.09	
Male	234/946	0.988 (0.970–1.007)	0.22	
Race/ethnicity				0.09
White	251/1015	0.976 (0.958–0.995)	0.01	
Others	218/692	1.000 (0.987–1.014)	0.96	
HTN				0.70
No	228/784	0.984 (0.966–1.003)	0.10	
Yes	241/923	0.990 (0.975–1.005)	0.19	
DM				0.52
No	318/1144	0.990 (0.977–1.004)	0.17	
Yes	151/563	0.978 (0.956–0.999)	0.04	
180-day mortality				
Age group				< 0.001
< 60 years	94/471	1.027 (1.005–1.051)	0.02	
Over 60 years	429/1236	0.965 (0.949–0.980)	< 0.001	
Sex				0.91
Female	257/761	0.983 (0.968–0.999)	0.04	
Male	266/946	0.982 (0.964–1.001)	0.06	
Race/ethnicity				0.08
White	290/1015	0.971 (0.953–0.989)	0.002	
Others	233/692	0.997 (0.982–1.012)	0.67	
HTN				0.59
No	259/784	0.978 (0.960–0.996)	0.02	
Yes	264/923	0.987 (0.971–1.002)	0.09	
DM				0.25
No	351/1144	0.988 (0.975–1.002)	0.08	
Yes	172/563	0.970 (0.949–0.991)	0.005	
1-year mortality				
Age group				< 0.001
< 60 years	105/471	1.019 (0.998–1.041)	0.08	
Over 60 years	479/1236	0.966 (0.952–0.981)	< 0.001	
Sex				0.84
Female	289/761	0.980 (0.965–0.996)	0.01	
Male	295/946	0.983 (0.965–1.000)	0.05	
Race/ethnicity				0.06
White	325/1015	0.970 (0.953–0.987)	< 0.001	
Others	259/692	0.996 (0.982–1.010)	0.58	
HTN				0.91
No	292/784	0.978 (0.962–0.995)	0.01	
Yes	292/923	0.984 (0.968–0.999)	0.04	
DM				0.29
No	382/1144	0.987 (0.973–1.000)	0.05	
Yes	202/563	0.970 (0.951–0.989)	0.002	

HRs were adjusted for age, gender, ethnicity, HTN, DM, HF, thrombolysis, thrombectomy, WBC, RBC, SBP, and SOFA

## Discussion

This retrospective study marks the first examining the correlation between TyG-BMI levels and ACM in critically ill stroke patients. Our findings identified significant L-shaped associations between TyG-BMI levels and long-term ACM, demonstrating that lower TyG-BMI levels relate to a heightened long-term ACM risk in this patient population within the United States context. While L-shaped correlations observed between TyG-BMI levels and short-term ACM did not achieve statistical significance. These insights could inform the development of clinical guidelines aimed at minimizing mortality among these patients. Accordingly, TyG-BMI emerges as a viable marker for stratifying and managing stroke patients during long-term follow-up care.

IR constitutes a standard feature of both metabolic syndrome and obesity. The global rise in obesity rates is directly linked to an increase in IR and related CVD occurrence and frequency [25]. The mechanisms by which IR can lead to stroke are multifaceted and include hyperinsulinemia and hyperglycemia, chronic low-grade inflammation, endothelial dysfunction, lipid abnormalities, HTN, and blood clotting abnormalities. IR, a documented key risk factor for stroke development and progression, can damage blood vessels and endothelium, reduce the ability of blood vessels to dilate, and alter the balance between clotting and anti-clotting factors in the blood, leading to atherosclerosis (hardening and narrowing of the arteries), increased blood pressure and a higher risk of clot formation or bleeding, ultimately causing stroke development and progression.

The TyG-i, introduced in 2008, has been acknowledged as a cost-effective, uncomplicated, and dependable substitute indicator for IR [26]. It has undergone validation and is strongly linked with stroke, acting as an independent stroke onset and progression predictor in hospitalized individuals [23, 27]. The BMI, commonly used to gauge obesity, shows a significant positive correlation with stroke incidence, as evidenced by multiple studies [28–30]. The combined TyG-BMI metric is a valuable tool for assessing IR [18, 21–24, 31], demonstrating its utility in evaluating major adverse cardiovascular event risk, particularly in patients having ST-elevation myocardial infarction (STEMI) and those with pre-HTN or HTN [32, 33]. Moreover, elevated TyG-BMI levels have been found to be related to an increased stroke incidence, though this relationship has primarily been documented within the Chinese population, highlighting the need for further research across different ethnicities to corroborate these findings [21–24].

The significance of the TyG-BMI in forecasting cardiovascular incidents and stroke risk is well-documented. Hou et al. have manifested that TyG-BMI and ACM possessed a U-shaped correlation in individuals suffering from CHD and HTN, thereby affirming the index's predictive accuracy for ACM within this demographic [34]. Further research underscores its utility, with one study indicating that TyG-BMI is a significant independent predictor of AF recurrence post-ablation, showcasing the highest predictive value among studied markers [35]. In intravenous thrombolysis-treated AIS patients, TyG-BMI has been identified as indicative of a negative prognosis [36]. Additionally, among patients receiving peritoneal dialysis, an elevated baseline TyG-BMI correlates with an increased ACM risk [37], reinforcing the index’s prognostic value across diverse medical scenarios. However, research on its connection with ACM in critically ill stroke patients remains scarce. Our investigation reveals that TyG-BMI can forecast long-term ACM in such patients, with a TyG-BMI and ACM L-shaped relationship being observed. This insight could be pivotal for identifying high-risk individuals in critical care settings, potentially aiding clinical trial designs. Subgroup analyses indicate that critically ill stroke patients, particularly those over 60 and White individuals, lower TyG-BMI levels exhibit higher ACM rates over 90-day, 180-day, and 1-year intervals and TyG-BMI is significant negative associated with ACM which is a protective factor. Conversely, ages under 60 years, the forest plots demonstrated that TyG-BMI is significant positive associated with ACM which is a risk factor. Furthermore, an elevated TyG-BMI appears to be beneficial for critically ill stroke patients who have non-HTN or DM, suggesting the index's broader applicability in managing and understanding stroke outcomes across diverse patient profiles. Our study shows that TyG-BMI is a highly effective clinical indicator for identifying critically ill stroke patients. Managing ICU-admitted critically ill patients is a significant subject and the primary focus of clinical practice. TyG-BMI, a readily available parameter upon ICU admission, might effectively assist clinicians in promptly identifying high-risk patients, hence reducing fatality rates and enhancing patient prognosis.

Given the robust evidence indicating that elevated TyG-BMI levels correlate with an increased risk of stroke [22–24], the discovery of an inverse relationship between TyG-BMI levels and mortality in our study may appear counterintuitive at first glance. This investigation centers on a cohort of patients who have not only experienced a stroke but are also critically ill. The health status and prognosis of this specific group diverge markedly from the general population that has not encountered a stroke, necessitating a nuanced interpretation of risk factors. Critically ill patients exhibit a spectrum of complex physiological alterations and metabolic dysregulations, potentially leading to a divergent pattern of risk factors compared to the general population. Furthermore, TyG-BMI serves as an indicator of insulin resistance and metabolic health. In the broader population, elevated TyG-BMI levels signal deteriorating metabolic health, thereby elevating the risk of cardiovascular incidents, including strokes. Conversely, among stroke survivors, diminished TyG-BMI levels may reflect severe malnutrition or metabolic exhaustion, conditions that are intimately linked with disease severity and an elevated mortality risk. Our findings corroborate the hypothesis of a significant negative correlation between TyG-BMI and ACM in critically ill stroke patients, aligning with previous studies [21, 38]. This alignment underscores the importance of TyG-BMI levels within the specific health trajectory of critically ill stroke patients, differentiating their risk profile from that of the general population.

Our study faces multiple constraints that merit attention. First, the retrospective nature and single-center design, relying on MIMIC-IV database-retrieved observational data, make the establishment of a definitive causal correlation challenging. Despite adjustments for numerous variables and conducting subgroup analyses, the potential impact of unaccounted confounders on our findings cannot be entirely ruled out such as subtypes of stroke, eliminations based on the National Institutes of Health Stroke Scale, stroke onset timing, and specific death causes being inaccessible within the utilized database. Second, the study's moderate sample size calls for validation through larger-scale cohort studies to reinforce our conclusions. Third, the initial blood glucose and lipid measurements were taken from patients upon their ICU admission, raising uncertainties about whether these readings were from fasting individuals. This ambiguity could affect the reliability of these variables in our analysis. Fourth, Despite the comprehensive analysis presented in this cohort, it is important to note that the study predominantly involved African-American and other racial groups within the United States. Consequently, the applicability of our results is primarily limited to the American context. Given this limitation, the extrapolation of our findings to predict critically ill stroke’s prognosis in other continents such as Europe, Asia, and Australia warrants further investigation to understand potential variations across different ethnic and geographical populations. Fifth, our investigation does not explore the biological mechanisms underpinning the TyG-BMI and ACM relation in critically ill stroke patients, leaving a gap in understanding the causative pathways. Additionally, the inability to determine the precise timing of stroke onset and identify the primary causes of death diminishes the clinical applicability of our findings. Addressing these limitations in upcoming studies could offer a more in-depth understanding of the TyG-BMI's role in predicting outcomes for critically ill stroke patients.

## Conclusion

In our study, lower TyG-BMI levels in critically ill stroke patients are significantly related to a higher risk of long-term ACM within the context of the United States. This finding suggests the potential of TyG-BMI as a marker for stratifying long-term risk in this patient population. However, it's crucial to note that this association was not observed for short-term ACM, indicating that the utility of TyG-BMI may be more pronounced in long-term outcome prediction. Additionally, our conclusion that TyG-BMI could serve as a reliable indicator for managing and stratifying stroke patients over the long term is preliminary. To confirm our findings and assess the universal applicability of TyG-BMI as a prognostic tool, it is crucial to conduct rigorously designed research across various populations.

### Supplementary Information

**Additional file 1:** **Figure S1.** Kaplan–Meier survival analysis curves for ACM and cumulative incidence of ICU (**A**), in-hospital (**B**), and 30-day (**C**) ACM. **Figure S2.** ROC of TyG-BMI for predicting ACM. **Figure S3.** Restricted cubic spline analysis of TyG-BMI and ICU (**A**), in-hospital (**B**), and 30-day (**C**) ACM. **Figure S4.** Forest plots of stratified analyses of TyG-BMI and ICU **(A)**, in-hospital **(B)**, and 30-day **(C)** ACM. **Table S1.** The baseline characteristics and outcomes between excluded and included participants**. Table S2.** Multivariable Cox proportional hazard models for short-term ACM. **Table S3.** Subgroup analyses of TyG-BMI and short-term ACM.

## Data Availability

Our contributions are detailed within the article and its Supplementary Material. For additional concerns regarding the research, please contact the corresponding author. The Supplementary Material is available online for reference.
