# Supplementary material for: Long-term survival in stroke patients: insights into triglyceride-glucose body mass index from ICU data
Source: Cardiovasc Diabetol. 2024 Apr 25;23:137. doi: 10.1186/s12933-024-02231-0 (PMC11046846; doi:10.1186/s12933-024-02231-0)

**Supplementary Materials**

Supplement to: Huang YW, Li ZP, Yin XS. Long-Term Survival in Stroke Patients: Insights into Triglyceride-Glucose Body Mass Index from ICU Data.

**Contents**

**Supplemental Table 1.** The baseline characteristics and outcomes between excluded and included participants.

**Supplemental Figure 1.** Kaplan-Meier survival analysis curves for ACM and cumulative incidence of ICU **(A)**, in-hospital **(B)**, and 30-day **(C)** ACM.

**Supplemental Figure 2.** ROC of TyG-BMI for predicting ACM.

**Supplemental Table 2.** Multivariable Cox proportional hazard models for short-term ACM.

**Supplemental Figure 3.** Restricted cubic spline analysis of TyG-BMI and ICU **(A)**, in-hospital **(B)**, and 30-day **(C)** ACM.

**Supplemental Table 3.** Subgroup analyses of TyG-BMI and short-term ACM.

**Supplemental Figure 4.** Forest plots of stratified analyses of TyG-BMI and ICU **(A)**, in-hospital **(B)**, and 30-day **(C)** ACM.

**Supplemental Table 1.** The baseline characteristics and outcomes between excluded and included participants.

| Variable | Include (n = 1707) | Exclude (n = 5796) | *P* value |
| --- | --- | --- | --- |
| Demographics |  |  |  |
| Age, years | 68 (58-78) | 72 (60-82) | < 0.001 |
| Men, n (%) | 946 (55.42) | 2986 (51.52) | 0.005 |
| Ethnicity, n (%) |  |  | < 0.001 |
| Asian | 56 (3.28) | 163 (2.81) |  |
| White | 190 (11.13) | 3721 (64.20) |  |
| Black | 1015 (59.46) | 506 (8.73) |  |
| Others | 446 (26.13) | 1406 (24.26) |  |
| Comorbidities |  |  |  |
| Hypertension, n (%) | 923 (54.07) | 3138 (54.14) | 0.96 |
| Diabetes mellitus, n (%) | 563 (32.98) | 1758 (30.33) | 0.04 |
| Heart failure, n (%) | 473 (27.71) | 1285 (22.17) | < 0.001 |
| Cardiac arrhythmias, n (%) | 784 (45.93) | 1908 (32.92) | < 0.001 |
| Acute myocardial infarction, n (%) | 28 (1.64) | 57 (.9834) | 0.02 |
| Peripheral vascular disease, n (%) | 160 (9.37) | 297 (5.124) | < 0.001 |
| Chronic obstructive pulmonary disease, n (%) | 118 (6.91) | 416 (7.177) | 0.71 |
| Chronic kidney disease, n (%) | 340 (19.92) | 1094 (18.88) | 0.33 |
| Hyperlipidemia, n (%) | 959 (56.18) | 2572 (44.38) | < 0.001 |
| Malignancy, n (%) | 320 (18.75) | 1264 (21.81) | 0.006 |
| Renal failure, n (%) | 1369 (80.20) | 3592 (61.97) | < 0.001 |
| Sepsis, n (%) | 1108 (64.91) | 2352 (40.58) | < 0.001 |
| Pneumonia, n (%) | 165 (9.67) | 167 (2.881) | < 0.001 |
| Liver disease, n (%) | 234 (13.71) | 697 (12.03) | 0.06 |
| Respiratory failure, n (%) | 676 (39.6) | 1135 (19.58) | < 0.001 |
| CCI | 6 (4-8) | 6 (4-8) | 0.004 |
| Vital sign |  |  |  |
| Mean blood pressure, mmHg | 86 (75-100) | 88 (77-100) | 0.02 |
| Systolic blood pressure, mmHg | 129 (113-149) | 134 (116-151) | < 0.001 |
| Diastolic blood pressure, mmHg | 69 (58-83) | 71 (60-83) | 0.08 |
| Mean heart rate, beats/min | 82 (72-96) | 81 (71-94) | 0.03 |
| Respiratory rate, times/min | 18 (15-22) | 18 (15-22) | 0.75 |
| SpO_2_, % | 99 (96-100) | 98 (96-100) | < 0.001 |
| Laboratory parameters |  |  |  |
| Red blood cell, 10^9^/L | 3.79 (3.20-4.32) | 3.84 (3.29-4.33) | 0.02 |
| Hemoglobin, g/L | 11.4 (9.6-13.0) | 11.6 (9.9-13.0) | 0.01 |
| Platelet, 10^9^/L | 191 (146-248) | 198 (153-252) | 0.002 |
| White blood cell, 10^9^/L | 11.0 (8.1-14.3) | 10.1 (7.6-13.6) | < 0.001 |
| Sodium, mmol/L | 139 (136-141) | 139 (136-142) | 0.19 |
| Blood urea nitrogen, mg/dL | 18 (13-28) | 18 (13-26) | 0.01 |
| Creatinine, mg/24h | 1.0 (0.7-1.4) | 0.9 (0.7-1.3) | < 0.001 |
| Potassium, mmol/L | 4.1 (3.7-4.5) | 4.0 (3.7-4.4) | 0.008 |
| AG, mmol/L | 14 (12-16) | 14 (12-16) | < 0.001 |
| Prothrombin time, s | 13.4 (12.1-15.6) | 12.9 (11.8-14.9) | < 0.001 |
| Activated partial thromboplastin time, s | 29.5 (26.3-34.7) | 29.1 (26.2-33.6) | 0.06 |
| International normalized ratio | 1.2 (1.1-1.4) | 1.2 (1.1-1.4) | < 0.001 |
| Scores |  |  |  |
| GCS | 15 (15-15) | 15 (14-15) | < 0.001 |
| SOFA | 1 (0-3) | 1 (0-2) | < 0.001 |
| SAPS-II | 35 (28-45) | 33 (26-42) | < 0.001 |
| SIRS | 3 (2-3) | 2 (2-3) | < 0.001 |
| OASIS | 33 (27-38) | 30 (25-36) | < 0.001 |
| APS-III | 41 (30-55) | 36 (27-48) | < 0.001 |
| Treatment |  |  |  |
| Vasopressors, n (%) | 735 (43.06) | 1363 (23.52) | <0.001 |
| Oxygen, n (%) | 1331 (77.97) | 3726 (64.29) | <0.001 |
| Thrombolysis, n (%) | 100 (5.86) | 268 (4.62) | 0.04 |
| Thrombectomy, n (%) | 192 (11.25) | 506 (8.73) | 0.002 |
| Stroke Type |  |  |  |
| AIS, n (%) | 1370 (80.26) | 4179 (72.10) | < 0.001 |
| ICH, n (%) | 434 (25.42) | 1553 (26.79) | 0.26 |
| SAH, n (%) | 116 (6.80) | 633 (10.92) | < 0.001 |
| Clinical Outcomes |  |  |  |
| LOS ICU, day | 5 (2-10) | 2 (1-4) | < 0.001 |
| LOS Hospital, day | 11 (6-20) | 7 (4-12) | < 0.001 |
| **Short-term all-cause mortality** |  |  |  |
| ICU mortality, n (%) | 211 (12.36) | 670 (11.56) | 0.37 |
| In-hospital mortality, n (%) | 300 (17.57) | 991 (17.10) | 0.64 |
| 30-day mortality, n (%) | 354 (20.74) | 1203 (20.76) | 0.99 |
| **Long-term all-cause mortality** |  |  |  |
| 90-day mortality, n (%) | 469 (27.48) | 1503 (25.93) | 0.20 |
| 180-day mortality, n (%) | 523 (30.64) | 1683 (29.04) | 0.20 |
| 1-year mortality, n (%) | 584 (34.21) | 1910 (32.95) | 0.33 |

**Supplemental Figure 1.** Kaplan-Meier survival analysis curves for ACM and cumulative incidence of ICU **(A)**, in-hospital **(B)**, and 30-day **(C)** ACM.


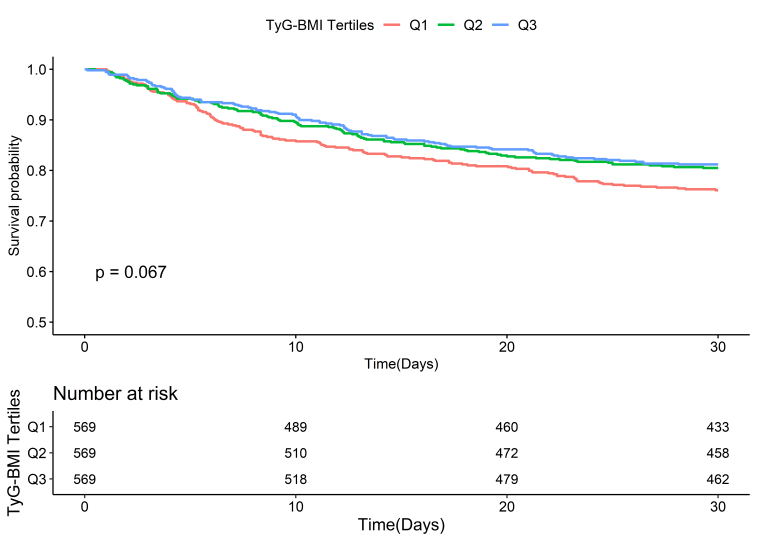


**Supplemental Figure 2.** ROC of TyG-BMI for predicting ACM.

**
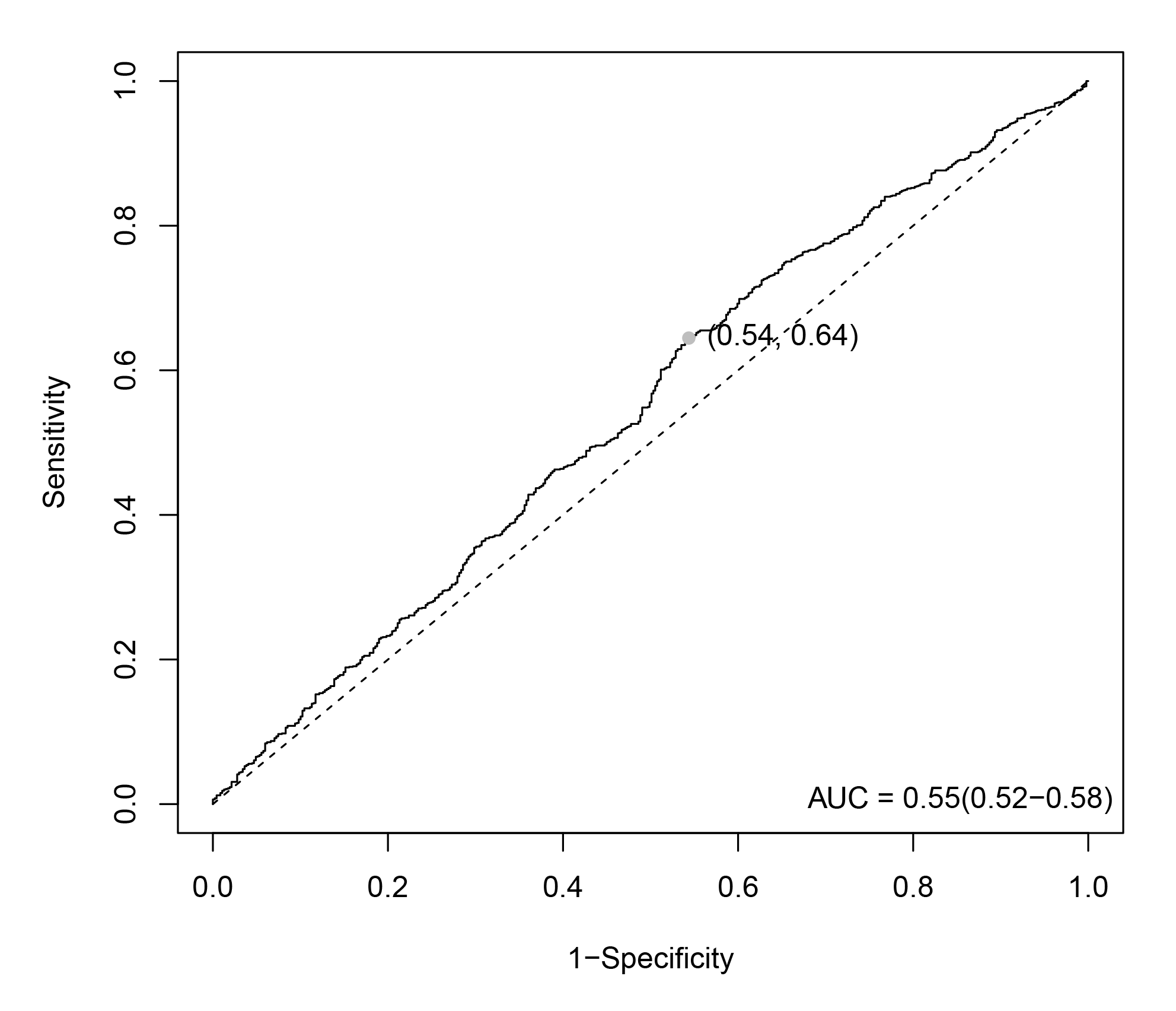

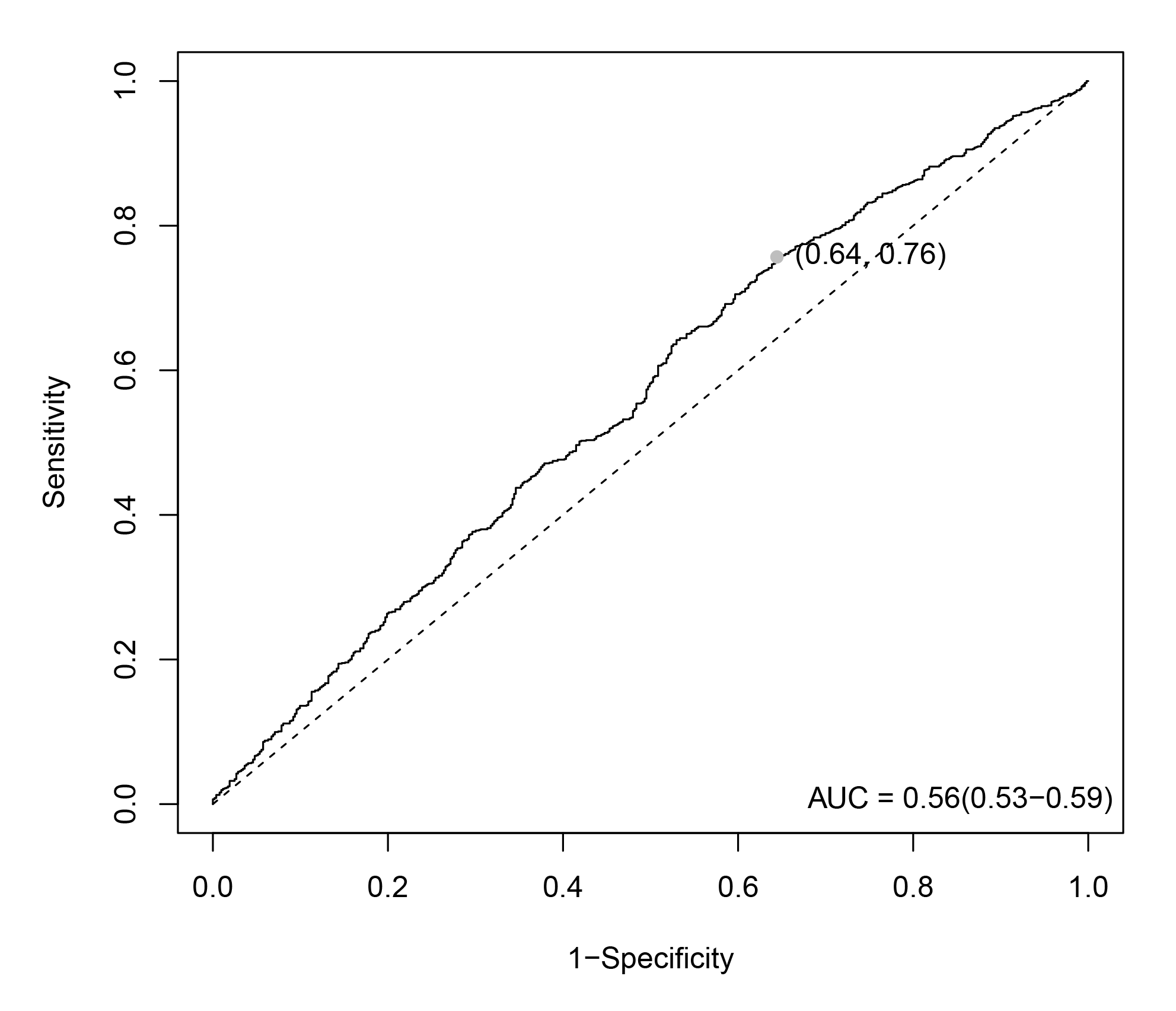

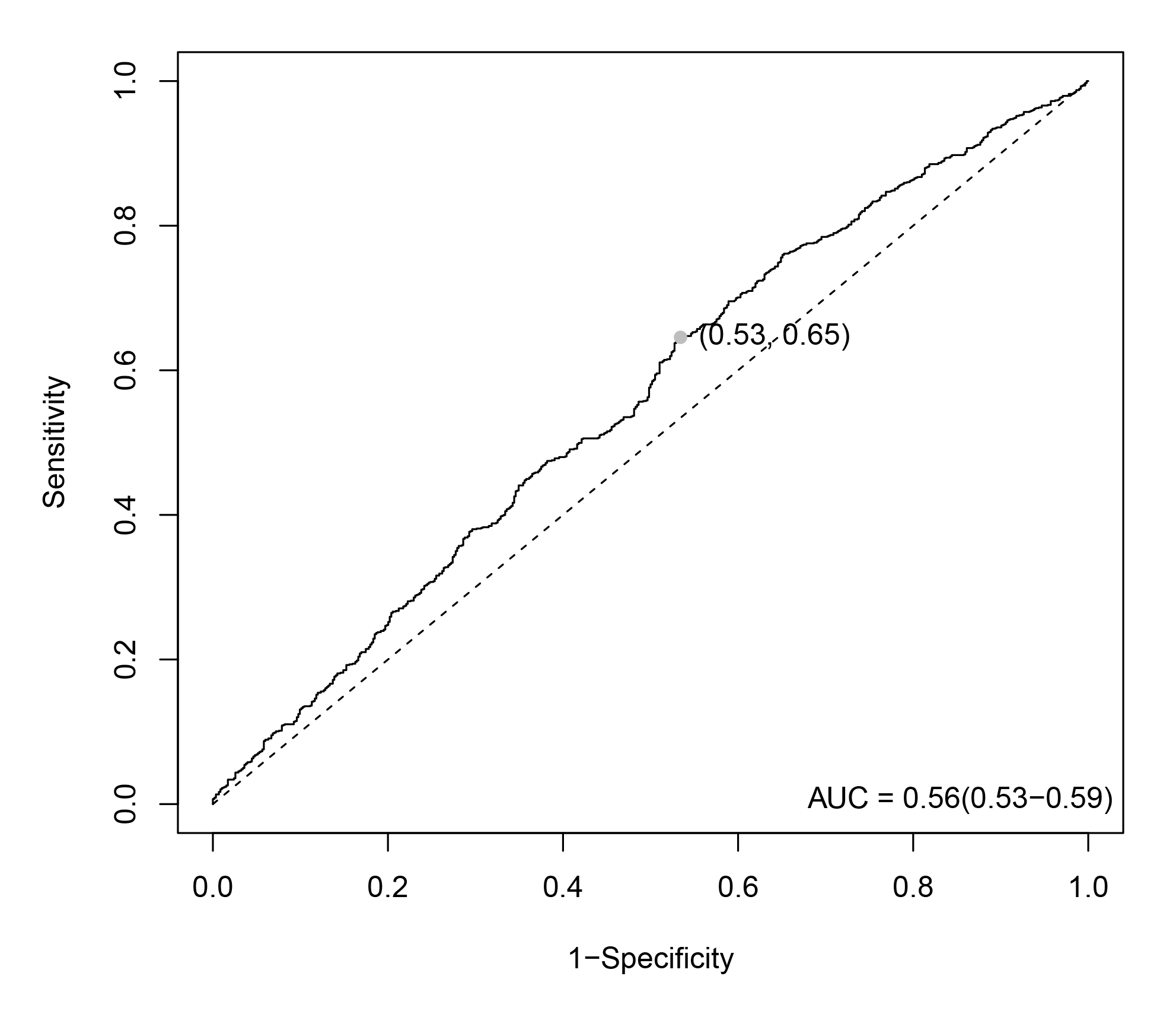
**

1. **day 180-day 1-year**

**
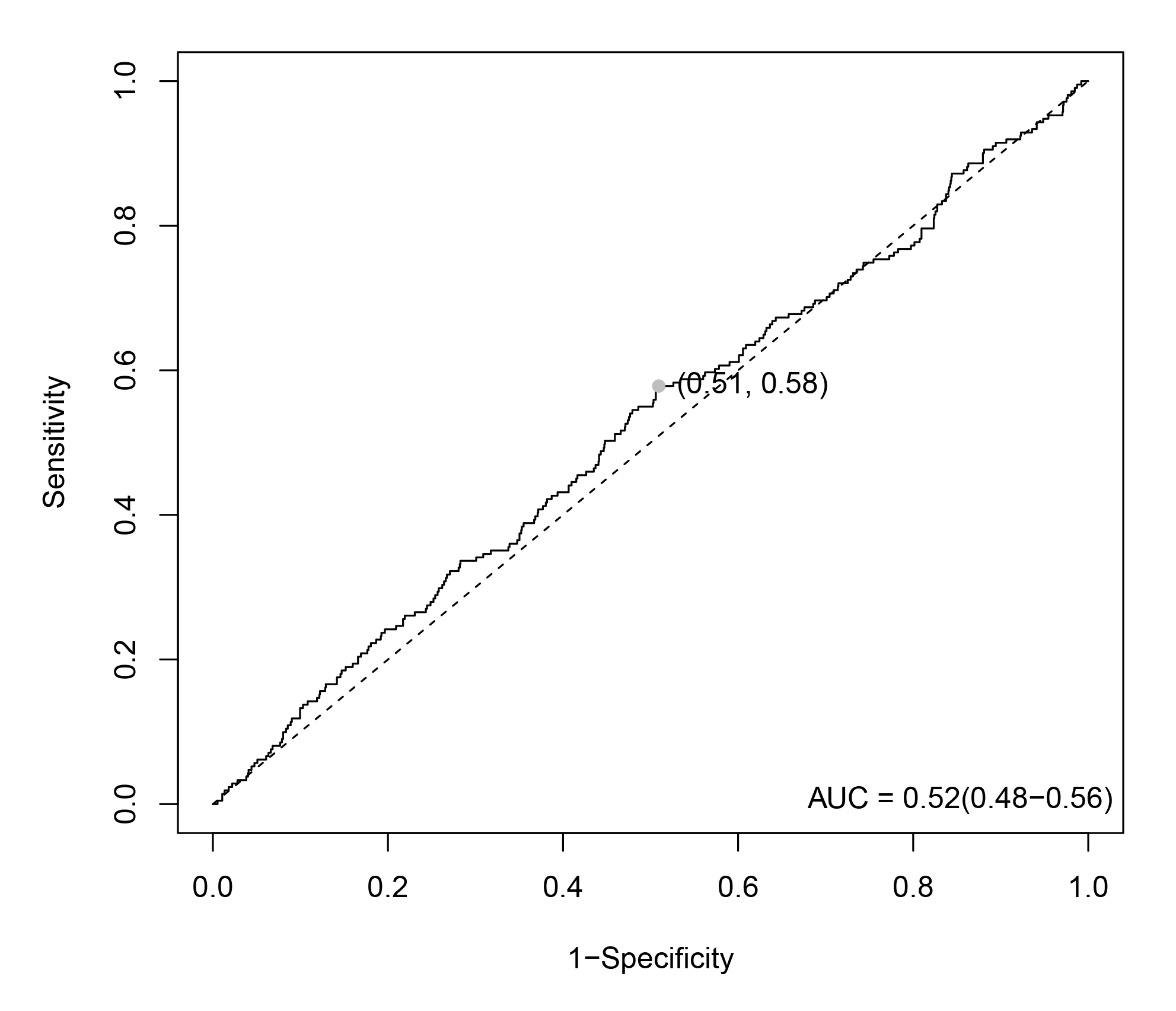

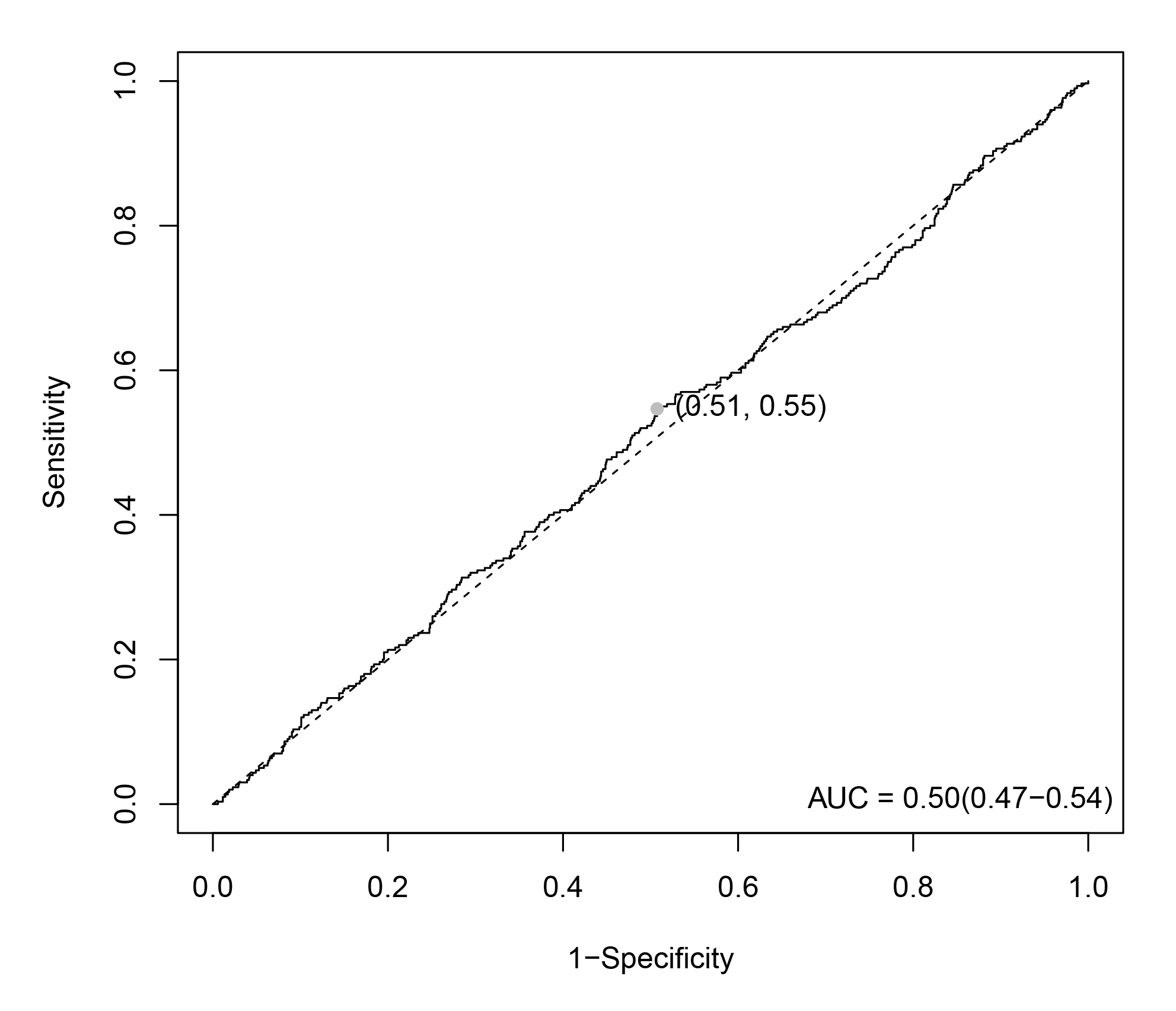

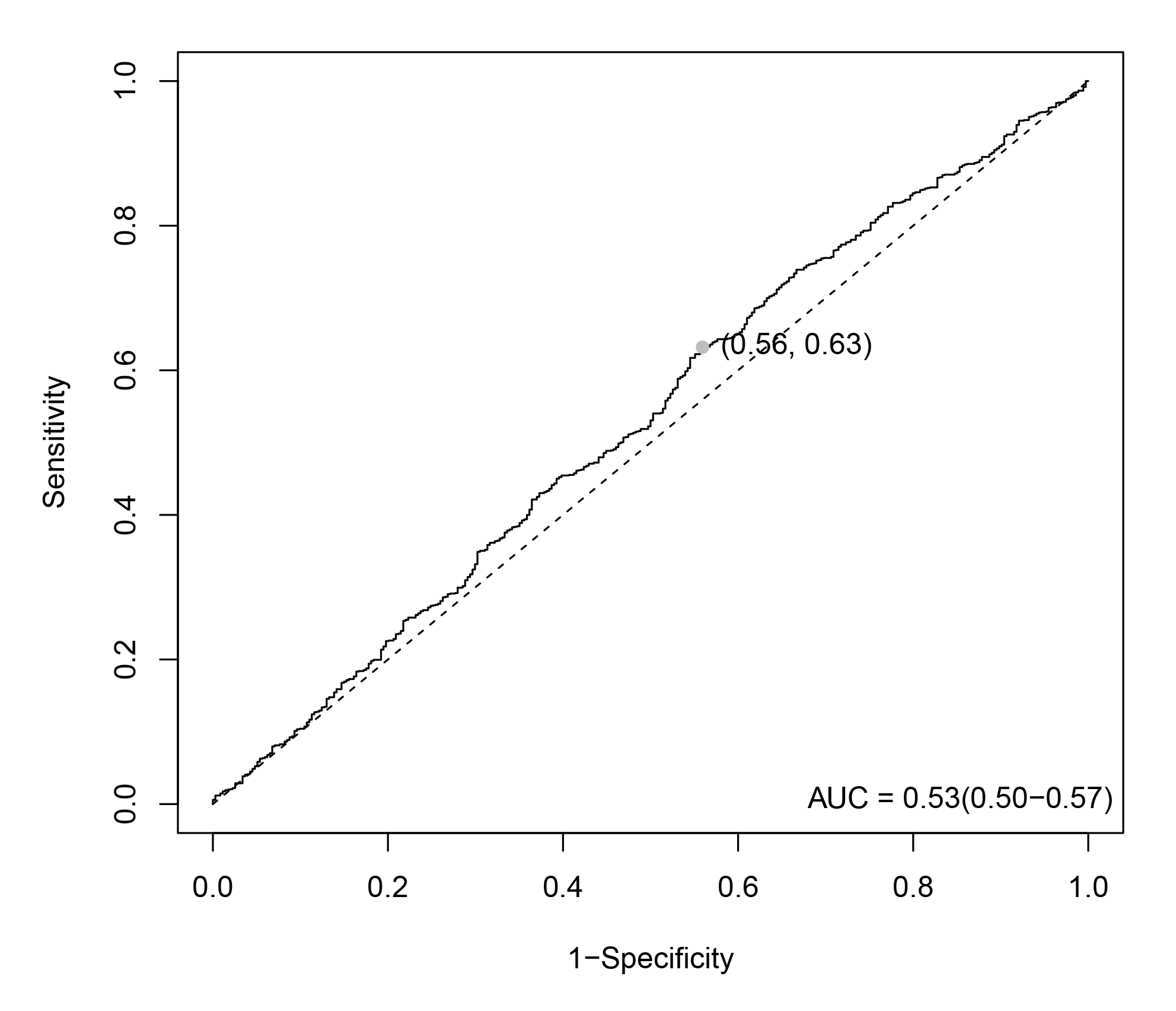
**

**ICU in-hospital 30-day**

**Supplemental Table 2.** Multivariable Cox proportional hazard models for short-term ACM.

| Short-term ACM | Model 1 | | Model 2 | | Model 3 | |
| --- | --- | --- | --- | --- | --- | --- |
|  | HR (95% CI) | *P* value | HR (95% CI) | *P* value | HR (95% CI) | *P* value |
| **ICU mortality** | | | | | | |
| TyG-BMI (tertiles) |  |  |  |  |  |  |
| Tertile 1 | Reference |  | Reference |  | Reference |  |
| Tertile 2 | 0.90 (0.65-1.26) | 0.56 | 0.93 (0.66-1.30) | 0.67 | 0.91 (0.55-1.28) | 0.58 |
| Tertile 3 | 0.82 (0.59-1.14) | 0.24 | 0.84 (0.60-1.18) | 0.31 | 0.78 (0.55-1.12) | 0.18 |
| *P* for trend |  | 0.27 |  | 0.34 |  | 0.36 |
| **Hospital mortality** | | | | | | |
| TyG-BMI (tertiles) |  |  |  |  |  |  |
| Tertile 1 | Reference |  | Reference |  | Reference |  |
| Tertile 2 | 0.91 (0.69-1.20) | 0.56 | 0.96 (0.72-1.27) | 0.75 | 0.94 (0.71-1.25) | 0.67 |
| Tertile 3 | 0.89 (0.68-1.18) | 0.24 | 0.94 (0.71-1.25) | 0.69 | 0.93 (0.70-1.25) | 0.64 |
| *P* for trend |  | 0.26 |  | 0.37 |  | 0.45 |
| **30-day mortality** |  |  |  |  |  |  |
| TyG-BMI (tertiles) |  |  |  |  |  |  |
| Tertile 1 | Reference |  | Reference |  | Reference |  |
| Tertile 2 | 0.80 (0.62-1.02) | 0.07 | 0.82 (0.64-1.06) | 0.13 | 0.81 (0.63-1.05) | 0.12 |
| Tertile 3 | 0.76 (0.59-0.98) | 0.03 | 0.80 (0.62-1.04) | 0.09 | 0.81 (0.62-1.06) | 0.13 |
| *P* for trend |  | 0.07 |  | 0.14 |  | 0.21 |

Model 1: Unadjusted;

Model 2: Adjusted age, gender, and ethnicity;

Model 3: Adjusted age, gender, ethnicity, hypertension, diabetes, HF, thrombolysis, thrombectomy, WBC, RBC, systolic blood pressure, and SOFA.

**Supplemental Figure 3.** Restricted cubic spline analysis of TyG-BMI and ICU **(A)**, in-hospital **(B)**, and 30-day **(C)** ACM.

**(A) (B) (C)**

**
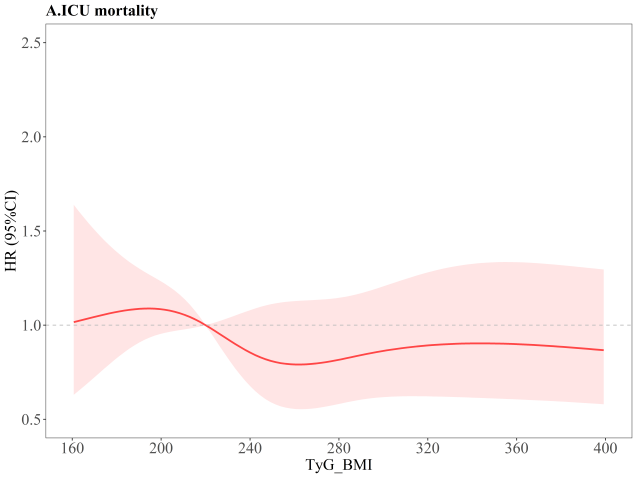

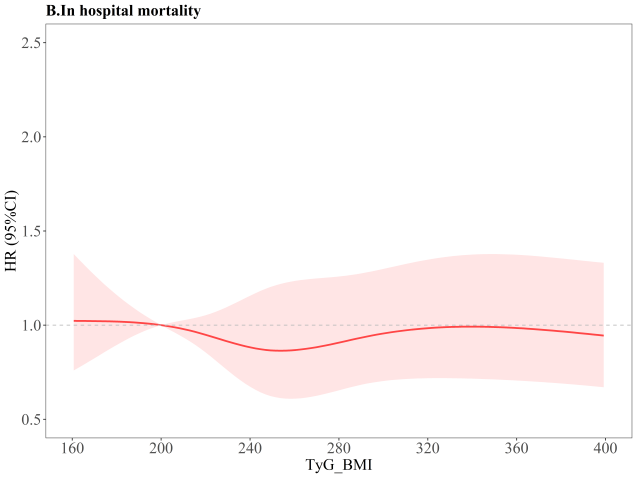

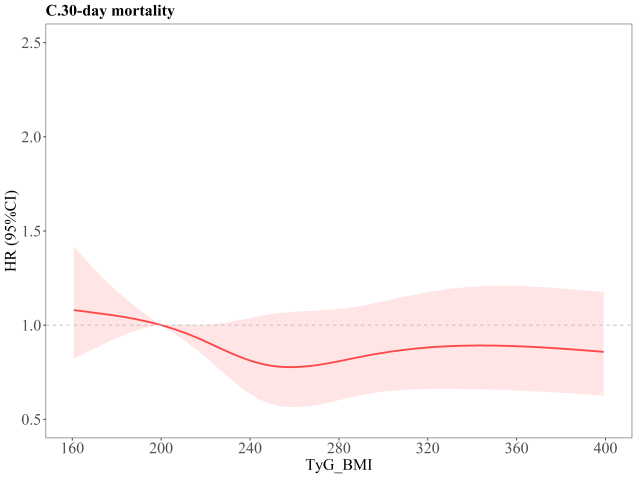
**

**Supplemental Table 3.** Subgroup analyses of TyG-BMI and short-term ACM.

| Variable | Cases/Total | HR (95% CI) | *P* value | *P* for interaction |
| --- | --- | --- | --- | --- |
| **ICU mortality** | | | | |
| Age group |  |  |  | 0.003 |
| <60 years | 105/471 | 1.031 (1.003-1.060) | 0.03 |  |
| Over 60 years | 479/1236 | 0.972 (0.948-0.996) | 0.02 |  |
| Sex |  |  |  | 0.64 |
| Female | 289/761 | 0.992 (0.973-1.012) | 0.45 |  |
| Male | 295/946 | 0.994 (0.969-1.020) | 0.20 |  |
| Ethnicity |  |  |  | 0.87 |
| White | 325/1015 | 0.993 (0.970-1.018) | 0.59 |  |
| Others | 259/692 | 0.997 (0.979-1.014) | 0.70 |  |
| HTN |  |  |  | 0.30 |
| No | 292/784 | 1.000 (0.977-1.023) | 0.97 |  |
| Yes | 292/923 | 0.988 (0.966-1.010) | 0.28 |  |
| DM |  |  |  | 0.96 |
| No | 382/1144 | 0.992 (0.975-1.010) | 0.40 |  |
| Yes | 202/563 | 0.999 (0.969-1.030) | 0.93 |  |
| **In-hospital mortality** | | | | |
| Age group |  |  |  | 0.003 |
| <60 years | 50/471 | 1.032 (1.007-1.059) | 0.01 |  |
| Over 60 years | 161/1236 | 0.984 (0.966-1.002) | 0.08 |  |
| Sex |  |  |  | 0.91 |
| Female | 100/761 | 0.994 (0.978-1.010) | 0.43 |  |
| Male | 111/946 | 0.999 (0.981-1.016) | 0.87 |  |
| Ethnicity |  |  |  | 0.66 |
| White | 92/1015 | 0.992 (0.973-1.012) | 0.44 |  |
| Others | 119/692 | 1.001 (0.987-1.015) | 0.90 |  |
| HTN |  |  |  | 0.60 |
| No | 101/784 | 0.999 (0.983-1.016) | 0.94 |  |
| Yes | 110/923 | 0.993 (0.977-1.010) | 0.44 |  |
| DM |  |  |  | 0.58 |
| No | 142/1144 | 0.997 (0.984-1.009) | 0.61 |  |
| Yes | 69/563 | 0.990 (0.964-1.017) | 0.46 |  |
| **30-day mortality** | | | | |
| Age group |  |  |  | < 0.001 |
| <60 years | 62/471 | 1.042 (1.015-1.069) | 0.002 |  |
| Over 60 years | 292/1236 | 0.975 (0.957-0.993) | 0.008 |  |
| Sex |  |  |  | 0.84 |
| Female | 175/761 | 0.989 (0.972-1.006) | 0.19 |  |
| Male | 179/946 | 0.997 (0.979-1.015) | 0.72 |  |
| Ethnicity |  |  |  | 0.24 |
| White | 181/1015 | 0.984 (0.964-1.004) | 0.12 |  |
| Others | 173/692 | 1.002 (0.988-1.016) | 0.76 |  |
| Hypertension |  |  |  | 0.86 |
| No | 161/784 | 0.994 (0.976-1.013) | 0.54 |  |
| Yes | 193/923 | 0.991 (0.975-1.007) | 0.28 |  |
| Diabetes |  |  |  | 0.60 |
| No | 246/1144 | 0.994 (0.981-1.008) | 0.40 |  |
| Yes | 108/563 | 0.985 (0.961-1.010) | 0.23 |  |

HRs were adjusted age, gender, ethnicity, hypertension, diabetes, HR, thrombolysis, thrombectomy, WBC, RBC, SBP, and SOFA.

**Supplemental Figure 4.** Forest plots of stratified analyses of TyG-BMI and ICU **(A)**, in-hospital **(B)**, and 30-day **(C)** ACM.

**(A)**  **(B) (C)**


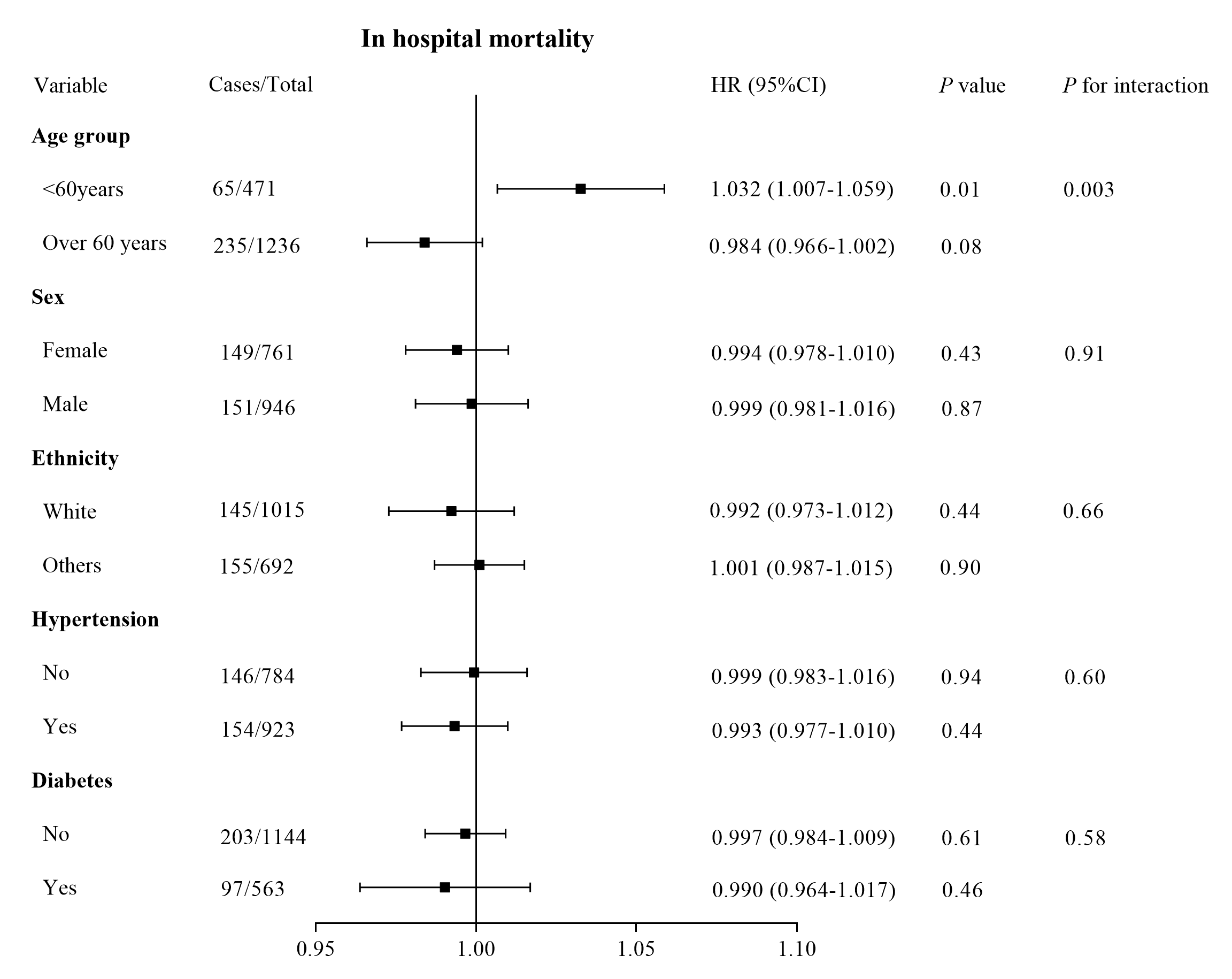

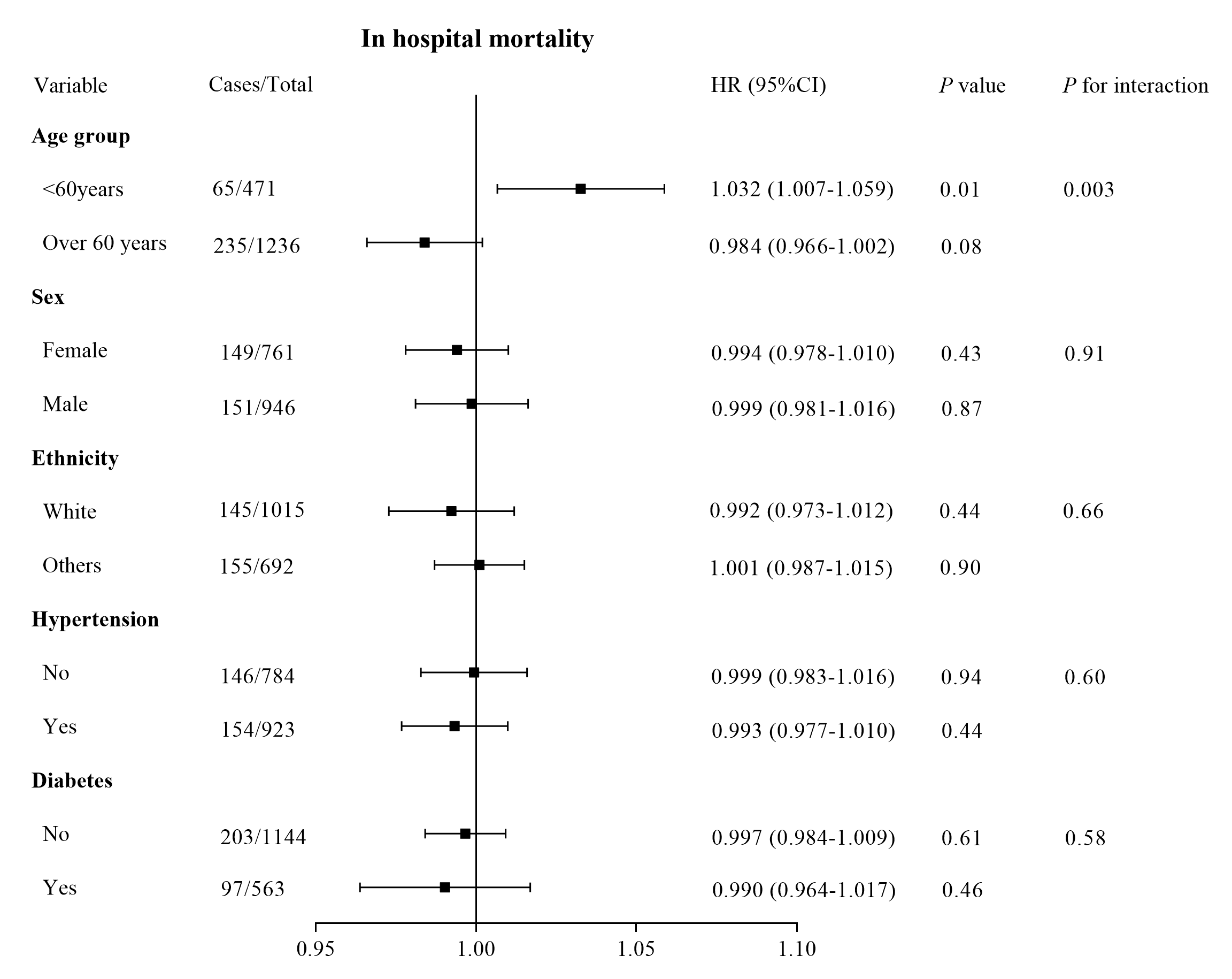

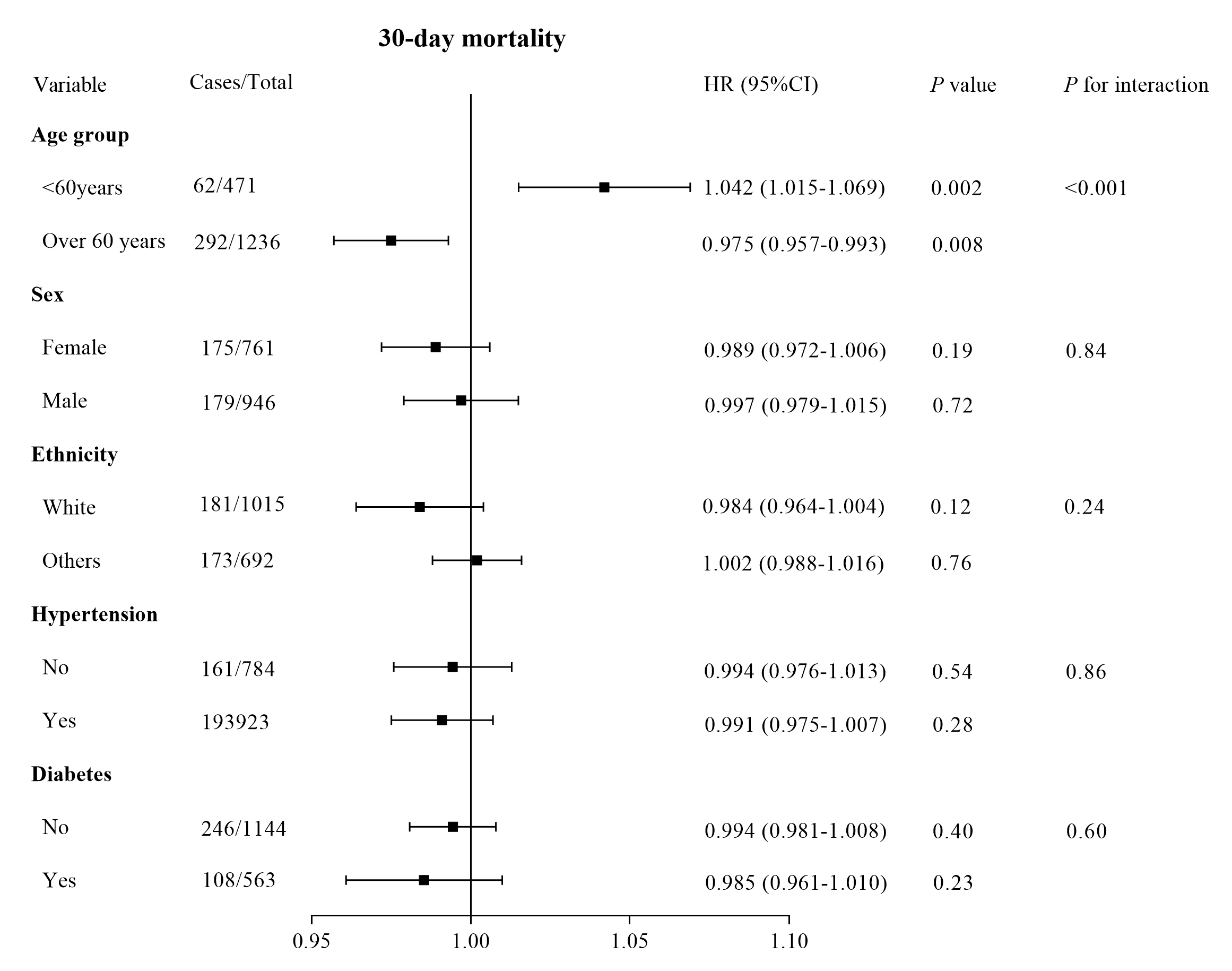

Supplement: Supplementary file 1 — Additional file 1: Figure S1. Kaplan–Meier survival analysis curves for ACM and cumulative incidence of ICU (A), in-hospital (B), and 30-day (C) ACM. Figure S2. ROC of TyG-BMI for predicting ACM. Figure S3. Restricted cubic spline analysis of TyG-BMI and ICU (A), in-hospital (B), and 30-day (C) ACM. Figure S4. Forest plots of stratified analyses of TyG-BMI and ICU (A), in-hospital (B), and 30-day (C) ACM. Table S1. The baseline characteristics and outcomes between excluded and included participants. Table S2. Multivariable Cox proportional hazard models for short-term ACM. Table S3. Subgroup analyses of TyG-BMI and short-term ACM. [file 12933_2024_2231_MOESM1_ESM.docx]
